# Training causes activation increase in temporo-parietal and parietal regions in children with mathematical disabilities

**DOI:** 10.1007/s00429-022-02470-5

**Published:** 2022-03-07

**Authors:** Mojtaba Soltanlou, Thomas Dresler, Christina Artemenko, David Rosenbaum, Ann-Christine Ehlis, Hans-Christoph Nuerk

**Affiliations:** 1 School of Psychology, University of Surrey, Guildford, UK; 2 Department of Psychology, University of Tuebingen, Tuebingen, Germany; 3 LEAD Graduate School & Research Network, University of Tuebingen, Tuebingen, Germany; 4 Department of Psychiatry and Psychotherapy, University Hospital of Tuebingen, Tuebingen, Germany

**Keywords:** Numerical cognition, Mathematical disabilities, Mental calculation, Arithmetic training, Functional near-infrared spectroscopy, fNIRS

## Abstract

While arithmetic training reduces fronto-temporo-parietal activation related to domain-general processes in typically developing (TD) children, we know very little about the training-related neurocognitive changes in children with mathematical disabilities (MD), who seek evidenced-based educational interventions. In a within-participant design, a group of 20 children (age range = 10–15 years old) with MD underwent 2 weeks of arithmetic training. Brain activation was measured using functional near-infrared spectroscopy (fNIRS) before and after training to assess training-related changes. Two weeks of training led to both behavioral and brain changes. Training-specific change for trained versus untrained (control) simple multiplication solving was observed as activation increase in the bilateral temporo-parietal region including angular gyrus and middle temporal gyrus. Training-specific change for trained versus untrained (control) complex multiplication solving was observed as activation increase in the bilateral parietal region including intraparietal sulcus, superior parietal lobule, and supramarginal gyrus. Unlike the findings of a similar study in TD children, 2 weeks of multiplication training led to brain activation increase in the fronto-parietal network in children with MD. Interestingly, these brain activation differences between the current findings and a recent similar study in TD children underlie a rather similar behavioral improvement as regards response time and accuracy after 2 weeks of training. This finding provides valuable insights into underlying mechanisms of mathematics learning in special samples and suggests that the findings in TD children may not be readily generalized to children with MD.

**Supplementary Information:**

The online version contains supplementary material available at 10.1007/s00429-022-02470-5.

## Introduction

Mathematical disability (MD) is a brain-based learning disorder affecting numerical and arithmetic abilities (De Smedt et al. 2019; Kaufmann et al. 2011). MD emerges at the early stages of development, affecting 3–6% of children and continues into adulthood (Kucian and von Aster 2015). It harms the career perspectives, mental health, and economic status of those diagnosed, and also puts a burden on society (Gross et al. 2009; Kaufmann et al. 2013). Surprisingly, we have little knowledge about the neural mechanisms of arithmetic processing in MD and the way these mechanisms change in the face of training. This knowledge will help us to further develop brain-based educational interventions directly derived from research in children with MD, whose brain responses might differ from typically developing (TD) children. Therefore, in the current study, we aim to investigate the neurocognitive mechanisms of arithmetic learning in children with MD.

The neural network of arithmetic processing consists of a widespread fronto-parietal network: the bilateral intraparietal sulcus (related to manipulation of magnitude) and the bilateral superior parietal lobule (related to visuospatial attention) and the left angular gyrus and hippocampus (related to verbal retrieval from long-term memory and attentional demands) and the prefrontal cortex (related to executive functions and cognitive demands) (Arsalidou et al. 2018; Dehaene et al. 2003; Klein et al. 2016). Studies in TD children reported consistent findings within the neural network of arithmetic processing with a rather high similarity across studies (Arsalidou et al. 2018; Peters and De Smedt 2018), whereas neuroimaging studies in children with MD solving arithmetic tasks provide divergent and seemingly contradictory findings.

One group of studies on arithmetic processing in children with MD reported higher activation (Davis et al. 2009; Rosenberg-Lee et al. 2015; Simos et al. 2008) and hyper-functional connectivity (Jolles et al. 2016; Michels et al. 2018; Rosenberg-Lee et al. 2015) in the fronto-parietal network as compared to TD children while solving arithmetic tasks. For instance, Davis et al. (2009) found higher activation in the bilateral precentral gyri and the right insula during simple addition in children with MD as compared to TD children, suggesting that children with MD rely on less advanced strategies to solve arithmetic problems. In a similar vein, Rosenberg-Lee et al. (2015) found higher activation in the right intraparietal sulcus, bilateral fusiform gyri, right visual cortex, and the left lingual gyrus during simple addition and subtraction in children with MD as compared to TD children (see also Simos et al. 2008). While showing higher brain activation, children with MD had worse behavioral performance in simple addition and subtraction tasks than that of TD children. Kucian and von Aster (2015) explain that children with MD overuse counting strategies and finger counting, have limited arithmetic fact retrieval, and experience difficulties with both procedural and conceptual knowledge; therefore, they overuse inefficient and compensatory strategies which leads to higher brain activation than seen in TD children. The conclusion from this group of studies is that children with MD have higher but inefficient brain activation, which is accompanied by poor behavioral performance (i.e., response time and accuracy) on mental arithmetic tasks as compared to TD children.

Another group of studies on the arithmetic abilities of children with MD observed reduced activation (Ashkenazi et al. 2012; Berteletti et al. 2014; Kucian et al. 2006; Peters et al. 2018; Schwartz et al. 2018) and structural connectivity (Rotzer et al. 2008; Rykhlevskaia et al. 2009) in the fronto-parietal network as compared to TD children during arithmetic problem solving. For instance, children with MD had reduced activation in the left inferior frontal gyrus, the left middle and superior temporal gyri, the right intraparietal sulcus, and the superior parietal lobule when completing simple multiplication tasks (Berteletti et al. 2014). The authors suggest impaired arithmetic mechanisms in both numerical- and language-related regions in the brains of children with MD. Ashkenazi et al. (2012) found reduced activation related to the complexity in addition in several regions, such as the intraparietal sulcus, superior parietal lobule, angular gyrus, and supramarginal gyrus in the right hemisphere, and bilaterally in the temporal and dorsolateral prefrontal cortex for children with MD as compared to TD children. Moreover, children with MD performed worse on the addition tasks than TD children. The conclusion from this group of studies is that arithmetic problem solving does not lead to the recruitment of the relevant neurocognitive resources in children with MD leading to poor behavioral performance.

These contradictory findings are not conclusive. It is unclear whether increased or decreased brain activation during arithmetic problem solving is an advantage or disadvantage in children with MD. More importantly, the question is how children with MD learn arithmetic and whether a behavioral improvement is accompanied by increased or decreased brain activation. While the first group of above-mentioned literature (i.e., higher but inefficient brain activation in children with MD) might suggest reduced but more efficient brain activation after training, the second group of literature (i.e., reduced and non-engaged necessary brain activation in children with MD) might suggest increased brian activation in the fronto-parietal network of mental calculation. By identifying these brain activation changes, the covert strategies that underly arithmetic problem solving will be disclosed to better understand the deficiencies in individuals with MD.

Intervention studies can provide this insight about changes in strategies and brain responses. So far, only very limited information is available about neuronal changes related to intervention (Iuculano et al. 2015; Kucian et al. 2011; Michels et al. 2018) or development (McCaskey et al. 2018, 2020) in children with MD. Of these studies, only one training study investigated neural activation changes during arithmetic learning in children with MD (Iuculano et al. 2015) similarly to our current study of arithmetic intervention. Iuculano et al. (2015) trained 15 children with MD for 8 weeks using one-on-one tutoring focusing on efficient counting strategies and arithmetic fact retrieval. Before training, they observed higher activation in the bilateral dorsolateral prefrontal and the left ventrolateral prefrontal cortex, the left intraparietal sulcus, the right fusiform gyrus, and bilateral insula during simple addition problem solving in children with MD as compared to age-matched TD peers. Thus, this training study supports the first group of literature discussed above in suggesting that there is higher brain activation in children with MD as compared to TD children. Interestingly, there were no differences in brain activation between the two groups of children after training as the over-engagement of the distributed brain activation was reduced in children with MD. This reduced activation over training manifests the existent inefficient widespread activation during simple arithmetic in MD, which is unnecessary, does not lead to appropriate performance on arithmetic tasks and therefore decreases after appropriate training.

The only existing neuroimaging study on arithmetic training in children with MD (Iuculano et al. 2015) is limited to simple calculation. Complex calculation differs from simple calculation in strategy use, and procedural and conceptual knowledge (Soltanlou et al. 2017a, b). Uncovering the neural mechanisms underlying complex calculation will help us to understand arithmetic learning beyond behavioral improvements in children with MD because these children struggle more with complex calculations. While behavioral training studies mainly support different interventional approaches, they do not answer the question of why children’s performance during arithmetic problem solving improves. Neuroimaging studies of arithmetic training provide more specific information about changes in the covert dysfunctions, which might be related to magnitude, cognitive, or language-related processes. These changes may not be clearly demonstrated in behavioral investigations because of the compensatory, but inefficient strategies that partially cover mathematical weaknesses. This knowledge would help us to develop better interventions that target particular mathematical weaknesses rather than an unspecific improvement in compensatory strategies in a child’s behavioral performance.

Therefore, in the present within-participant study, we investigate neural activation changes before and after 2 weeks of simple and complex multiplication training in children with MD. This study is built upon on our recent multiplication training in TD children (Soltanlou et al. 2018a, b), with a very similar procedure. We set out to examine whether the same training would lead to similar or different brain responses in children with MD. Moreover, by training both simple and complex arithmetic problems, we can extend the findings by Iuculano et al. (2015) that trained only simple arithmetic problems, and also test for the difficulty-related modulation of neural activity (Ashkenazi et al. 2012). The difficulty-related modulation of neural activity is expected as distinguishable brain responses to simple and complex calculation. Ashkenazi et al. (2012) reported no distinguishable neural activation patterns during simple and complex addition in children with MD in a single-session measurement, which is usually observed in TD children (Soltanlou et al. 2017a, b). Additionally, while simple multiplication is mainly solved via retrieval strategy in TD children, it may not necessarily be true in children with MD. Therefore, simple multiplication (specially larger than 5) may still rely on procedural strategies. Lastly, the task of interest in the current study is complex multiplication because in our previous study in TD children, we observed training-related changes only in complex but not simple multiplication (Soltanlou et al. 2018b). However, we are not sure whether complex multiplication would be too difficult for our children with MD that have the risk of drop-out, insufficient number of trials and corresponding fNIRS data. Therefore, we train both simple and complex multiplication in the current study.

Training-induced changes is evaluated using functional near-infrared spectroscopy (fNIRS), a well-suited technique for children (Soltanlou et al. 2018a, b; Soltanlou and Artemenko 2020). We predict that children with MD exhibit shorter response times and make fewer errors on both simple and complex multiplication tasks after training. Following Iuculano et al. (2015), we expect an overall reduced fronto-parietal activation after 2 weeks of training on simple multiplication, particularly in the right hemisphere, which is less engaged in advanced retrieval strategies. This activation reduction is mostly expected in the prefrontal regions that support procedural processes of mental calculation. Training would lead to less demands on these processes that are expected to be more automatized. However, this increased automatization might lead to an activation increase in the temporo-parietal regions such as the left angular gyrus (e.g., Rivera et al. 2005). Additionally, training might improve the capability of mental number manipulation that is associated with activation increase in the parietal regions, especially in the bilateral intraparietal sulcus. Concerning complex multiplication, two predictions can be derived from the two groups of literature discussed above. Based on the first literature group, which argued that children with MD have higher neural activation as compared to TD children, we would expect reduced fronto-parietal activation after training in children with MD, which will be more similar to TD children. This prediction is in line with the findings on complex multiplication tasks for TD children (Soltanlou et al. 2018b). According to the second literature group, which reported lower neural activation in children with MD, we would expect increased fronto-parietal activation after 2 weeks of complex multiplication training in children with MD. However, similar to our predictions in training of simple multiplication, we might observe an activation reduction in the frontal regions, but activation increase in the parietal regions (see Zamarian et al. 2009 for review of findings in adults). Therefore, while we have a directional hypothesis for simple multiplication training (i.e., reduced brain activation after training), there is no directional hypothesis for complex multiplication training.

Additionally, we expect differentiable brain activation between simple and complex multiplication (i.e., the difficulty-related modulation of neural activity) after training, like the complexity-related brain activation seen in TD children. Note that since the current study is a follow-up of our recent multiplication training in TD children (Soltanlou et al. 2018b), we will descriptively compare the current findings with the findings of that study as a control group.

## Materials and methods

### Participants

Twenty-five children with MD participated in the study. Five children were excluded: two did not finish the study, two because of technical problems with the fNIRS recording, and one because of only wrong answers in two conditions. Hence, 20 children (8 girls; age = 12.30 ± 1.13 years old; age range = 10.42–15.00 years old) were included in the analyses. The children were from grades 4–8 (grade 4:1, grade 5:3, grade 6:9, grade 7:4, grade 8:3). All children, except two, were right-handed. They had a normal or corrected-to-normal vision. All procedures of the study were in line with the latest revision of the Declaration of Helsinki and were approved by the ethics committee of the University Hospital of Tuebingen.

In a screening session, math ability was assessed with the Basisdiagnostik Mathematik für die Klassen 4–8 (BASIS-MATH 4–8, Moser Opitz et al. 2010). It is a standard screening test in German for all children in grades 4–8 that can identify numerical deficiencies independent of age and grade. The test evaluates basic mathematical knowledge, which is achieved in primary school, including basic arithmetic operations (i.e., mental arithmetic and written procedures), understanding of the decimal place-value system, counting, number line estimation, and word problems.

The critical threshold is the total raw score of 70 ± 3. This test uses raw scores—rather than standardized scores based on norms—so that any child who scored above the cut-off score of 73 was considered to be in the normal range of math abilities and was not included in the current study. A score of below 67 indicates that children did not understand basic classroom math content; 15 children in the current sample scored below 67. A score between 67 and 73 belongs to the tolerance zone indicating that children are at risk of difficulties to understand basic classroom math content; 5 children in the current sample scored between 67 and 73. The cut-off score of 73 in the current study thus includes children with math disabilities and being at risk of math difficulties. Since the recruitment was specific for children with MD, mainly parents and some teachers, who subjectively considered the child in their care to have math difficulties, approached us. Three children had a clinical diagnosis of dyscalculia, and one had attention-deficit/hyperactivity disorder. Children and their parents gave written informed consent and received an expense allowance for their participation.

### Characteristics of participants

A few neuropsychological tests were utilized to find the characteristics of the sample (see Table 1). Two subtests (similarities and matrix reasoning) of the German Wechsler IQ test (Hamburg–Wechsler-Intelligenztest für Kinder-IV, Petermann et al. 2007) were utilized to assess intelligence. According to the literature (Kucian and von Aster 2015), MD is not influenced by general intelligence, and children with an average or below-average level of intellect experience similar difficulties with arithmetic learning (Ehlert et al. 2012). The number of correct answers was transformed to IQ scores (*M* = 100, *SD* = 15).

**Table 1:** Neuropsychological data

	*Mean*	*SD*	*Min–Max*
Verbal IQ (similarities)	102	15.30	65–125
Non-verbal IQ (matrix reasoning)	95.5	20.90	55–120
Verbal short-term memory (letter span—forward)	4.5	0.83	3–6
Verbal working memory (letter span—backward)	3.4	1.00	3–5
Visuospatial short-term memory (Corsi—forward)	5.2	0.83	4–7
Visuospatial working memory (Corsi—backward)	5.0	0.89	3–7
Reading* (Salzburger reading test)	83.1	13.40	62–105
Mathematics knowledge (BASIS-MATH)	58.7	10.50	25–73

*N* = 20 (but 19 for the letter span—backward, and 18 for the reading test)

*Two children had a reading score of below 62, which suggests they are at risk of having a reading problem. Because the test does not provide a norm for the scores below 62, we did not include their reading score here

Verbal short-term and working memory, and visuospatial short-term and working memory were assessed using the letter span test and the block tapping test (Corsi 1973), respectively. In the verbal memory task, children were asked to recall spoken sequences of consonant letters (one letter per second). The test started with sequences of three letters, which were increased by one if the child correctly recalled at least two out of three sequences. In the visuospatial memory task, the child was asked to point to cubes in the same order as the experimenter. The procedure was the same as in the letter span test. These forward spans were considered to represent short-term memory, while backward spans (i.e., inverse recall) were considered to represent working memory in each test. Note that the score of one child was missing for the backward letter span test score (not included in Table 1).

To test the reading ability of the children, the Salzburger reading test for grades 2–9 (Salzburger Lese-Screening für die Schulstufen 2–9, Form A1; Wimmer and Mayringer 2014) was used. It contains 100 short sentences and children were asked to verify whether each sentence is grammatically correct or not. They had 3 min to evaluate as many sentences as they could. The number of correct answers was transformed to reading scores.

### Procedure

**Fig. 1:**
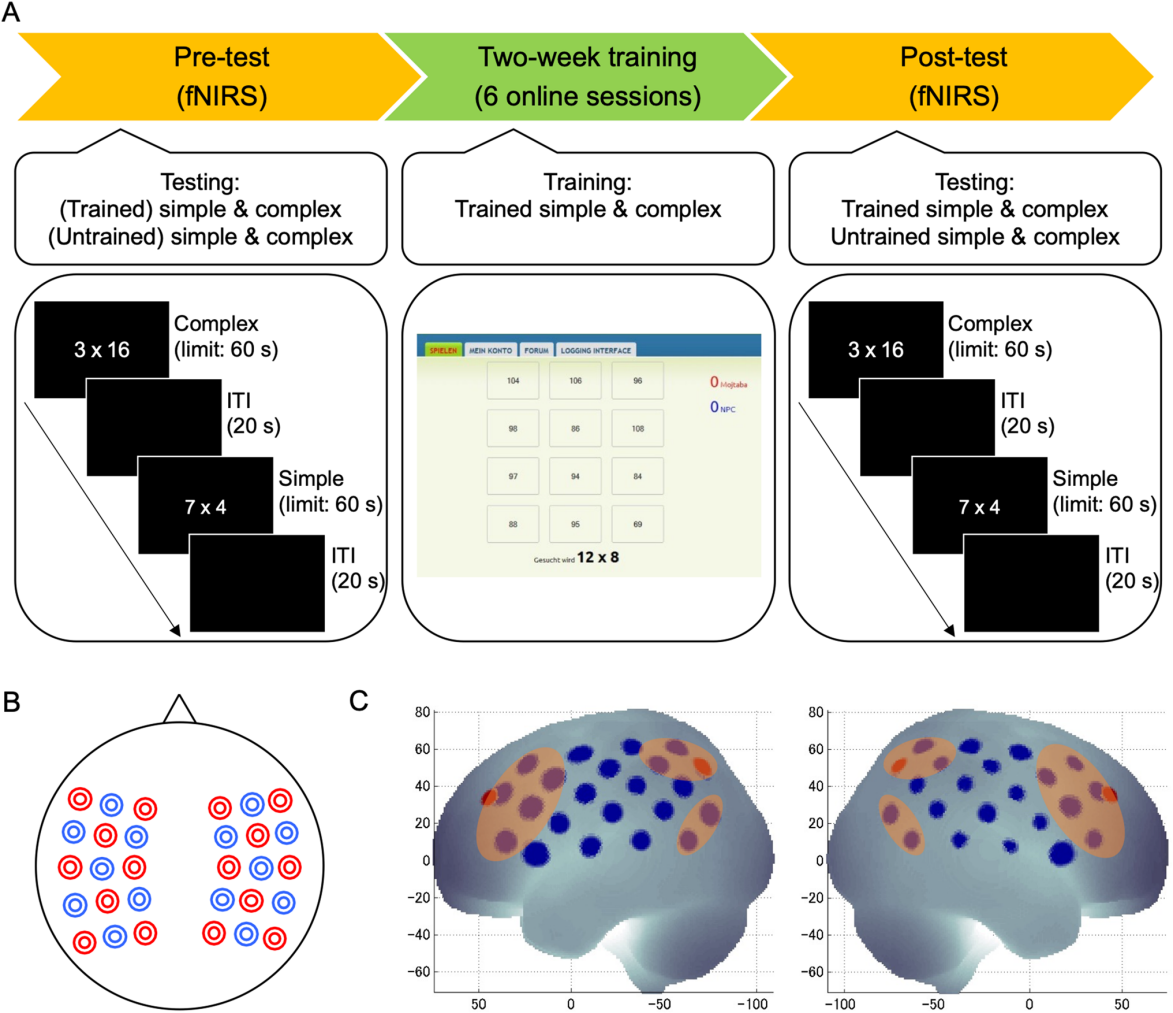
**A** Procedure of the study showing the pre-post design with fNIRS recordings and the online training of multiplication. **B** Schematic positions of fNIRS optodes. Red circles indicate emitters and blue circles indicate detectors in the two 3 × 5 arrays. **C** fNIRS channels layout (by Minako Uga). Blue circles indicate the fNIRS channels projected on the brain surface. Red circles indicate P3/P4, and F3/F4 positions—according to the international 10–20 system—projected on the brain surface. Channels included in the parietal, frontal, and temporo-parietal ROIs are marked by the orange ovals (see also Artemenko et al. 2018)

In a within-participant experiment, we measured behavioral performance and brain activation of children with MD during multiplication problem solving before and after training (Fig. 1A). The experiment was conducted in a light-attenuated room, where all the lights were turned off before starting the experiment. The experiment began with four practice trials. The multiplication problems were presented on the screen and the children were instructed to speak their answers as quickly and accurately as possible and then to immediately press the space-key (Fig. 1A). The experimenter, who was sitting behind the child, wrote down the answers on a sheet. Each problem was randomly presented once during the pre-test and the post-test. Each problem was presented until keypress or for a maximum duration of 60 s, and was followed by 20 s of rest (i.e., inter-trial interval; Fig. 1A). Note that these self-paced responses only appear as a variation in response times but not a variation in the number of trials. Each child solved exactly 32 problems (8 problems per condition) in each measurement time (pre- and post-test). No feedback was given during the experiment.

After the pre-test, six similar training sessions were performed through an online learning platform (Soltanlou et al. 2017b) at home for 2 weeks. In the post-test, children were measured again to evaluate training effects. The problems were identical for each condition in pre- and post-test sessions. The experiment was run using the software Presentation (version 16.3, NeuroBehavioral Systems, Inc., Berkeley, USA). This study was part of a combined fNIRS-EEG project, and only the fNIRS findings are reported here.

### Neuroimaging task

In the present study, 16 simple and 16 complex multiplication problems were used, the same as in our training study in TD children (Soltanlou et al. 2018b). Half of the problems in each set were allotted for training, and the other, closely matched half were used as untrained problems in the pre- and post-test only, resulting in four conditions: trained simple, untrained simple, trained complex, and untrained complex (for the list of the items see Supplementary Material, Table S7). Simple problems (e.g., 3 × 7) included two single-digit operands (range 2–9) with a two-digit solution (range 12–40). Complex problems (e.g., 4 × 19) included a two-digit operand (range 12–19) multiplied by a single-digit operand (range 3–8) with a two-digit solution (range 52–98). In half of the items in each condition, the first operand was larger than the second operand.

### Interactive online training

The training was done using an online learning platform (Soltanlou et al. 2018b, 2017b), which allowed for at-home training. The children participated in six training sessions over 2 weeks in their homes (Fig. 1A). Children were trained only for the trained simple and trained complex multiplication sets and not for the untrained sets. The multiplication problems within each condition were presented randomly in each run, for a total of five runs in each training session (i.e., a total of 80 problems per session). Each problem was individually presented along with 12 different choices including the correct solution. Detailed description of the choices can be found elsewhere (Soltanlou et al. 2018b). Response intervals for simple problems ranged randomly from 7 to 20 s, jittered by 1.3 s, and for complex problems from 15 to 45 s, jittered by 3 s. In an interactive competition, the computer displayed the correct solution whenever the child did not respond within the response interval. To provide feedback on performance and to increase motivation, the scores of the child and the computer were shown on the right side of the screen (see also Soltanlou et al. 2018b). Both the child and the computer received one point for each correct answer and one point was deducted for each incorrect answer. The problem was presented until the child responded correctly or correct answer was given. To create a more realistic competition and to increase participant motivation, the computer responded incorrectly for 30% of the problems. Children were instructed to solve the problems as quickly and accurately as possible.

### fNIRS recording and preprocessing

fNIRS data were collected with the ETG 4000 Optical Topography System (Hitachi Medical Corporation, Tokyo, Japan) using two wavelengths of 695 ± 20 nm and 830 ± 20 nm to measure the absorption changes of oxygenated (O_2_Hb) and deoxygenated (HHb) hemoglobin, according to the modified Beer–Lambert law. The data were recorded with a 10-Hz sampling rate, the fixed inter-optode distance was 30 mm. Using two 3 × 5 arrangements of optodes (8 emitters and 7 detectors each, Fig. 1B) in an elastic combined fNIRS-EEG cap (Brain Products GmbH, Herrsching, Germany) resulted in 22 measurement channels for each hemisphere (Fig. 1C). The correspondence of the fNIRS channels to the underlying cortical regions was estimated based on a virtual registration method (Rorden and Brett 2000; Singh et al. 2005; Tsuzuki et al. 2007) and labeled according to the automated anatomical labeling (AAL) atlas (Tzourio-Mazoyer et al. 2002). For more details see Soltanlou et al. (2017a).

Continuous changes in the concentration of O_2_Hb and HHb were recorded for all channels during the measurements. These changes occur through neurovascular coupling in response to cortical activation. Data were preprocessed and analyzed with custom MATLAB routines (The MathWorks, Inc., USA). The continuous signals were bandpass filtered with 0.01–0.2 Hz to remove long-term drift of baseline, and high-frequency cardiac and respiratory activities (Haeussinger et al. 2014; Sasai et al. 2011; Scholkmann et al. 2014; Tong and Frederick 2010). The remaining noisy channels (7.5%) were detected by visual inspection for each participant and interpolated using the average signal of surrounding channels. Most of these channels were located on bilateral temples and were not included in the regions of interest (ROIs; see below). Incorrect and missing trials (10.5%) were excluded. To address possible motion artifacts, which is particularly important with children, and to reduce non-evoked systemic influences (Haeussinger et al. 2014; Scholkmann et al. 2014), we used the correlation-based signal improvement (CBSI) method (Cui et al. 2010), as one of the recommended artifact correction methods in fNIRS signal processing (Brigadoi et al. 2014; Fishburn et al. 2019). The CBSI time course, which is calculated based on the assumed negative correlation between concentrations of O_2_Hb and HHb, was used for further analysis. Using a data-driven approach based on the grand average of the fNIRS time series of all correctly solved trials across all channels, participants, and conditions, the amplitude of 10 s after stimulus onset (i.e., 0–10 s) was averaged for each channel, participant, and condition. The grand average revealed that the rising signal came back to the baseline at 10 s after the stimulus onset. This average was baseline-corrected using the 5 s before each respective trial and used for the analysis.

### Analysis

#### Behavioral

Response times (RTs) were defined as the time interval from problem presentation until participant keypress after the verbal answer in the pre- and post-measurements. Median RTs were calculated based on correct responses only (89.5% of problems across pre- and post-test). The error rate was defined as the proportion of incorrect and missing responses to the total number of presented trials. Separate repeated-measures analyses of covariance (rmANCOVAs) were conducted for median RTs and error rates, and for simple and complex multiplication. Each 2 × 2 rmANCOVA comprised the within-factors of measurement time (pre- versus post-test), and training (trained versus untrained) and grade as a continuous covariate because of its rather wide range. According to the literature, having a greater amount of education makes it easier to solve arithmetic problems, and different educational levels lead to differences in performance and brain activation patterns (Artemenko et al. 2018; McCaskey et al. 2018; Ranpura et al. 2013). We further conducted correlation analyses to test whether children’s performance in the pre-test predicts the training effect (i.e., changes in RT and error rate over time by calculating the difference between these variables in pre- and post-test).

#### fNIRS

In the first step, ROIs were defined within the neural network for arithmetic processing. They consisted of the frontal (bilateral middle frontal gyrus and inferior frontal gyrus), parietal (bilateral intraparietal sulcus, superior parietal lobule, and supramarginal gyrus), and temporo-parietal (bilateral angular gyrus and middle temporal gyrus) cortices (Fig. 1C). The 2 × 2 × 2 rmANCOVA comprised the within-factors of measurement time (pre- versus post-test), training (trained versus untrained), hemisphere (left versus right), and grade as a covariate. The rmANCOVAs were conducted for each ROI, separately for simple and complex multiplication.

In the next step, to test for the difficulty-related modulation of neural activity due to multiplication training, a global analysis was conducted. All 44 channels covering both hemispheres were defined as one ROI. The 2 × 2 rmANCOVA comprised the within-factors of measurement time (pre- versus post-test), complexity (simple versus complex), and grade as a covariate. The statistical analyses were completed using R (R Core Team 2018) and jamovi (Version 1.1.9) (2019).

## Results

### Behavioral

#### Simple

**Fig. 2:**
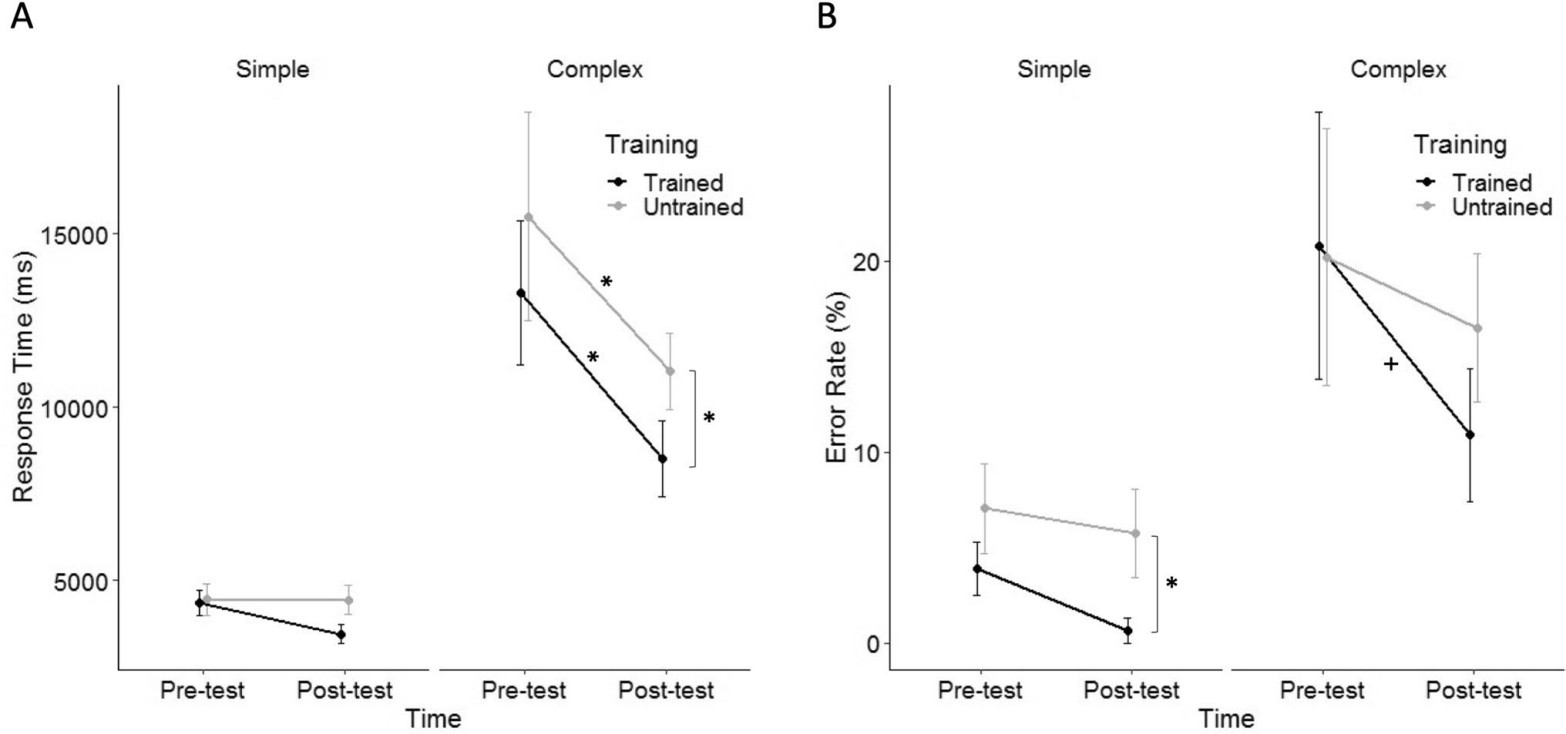
**A** Median RT and **B** Error rate for simple and complex multiplication pre-test and post-test after training on a portion of problems. Error bars depict 1 standard error (SE*)* of the mean. For simplicity, the values in the figure are not adjusted for the covariate. *p < 0.05; + *p* < 0.1

The rmANCOVA of median RT for simple multiplication revealed no significant effects (Fig. 2A). The rmANCOVA of error rate revealed a significant main effect of training [*F*(1,18) = 6.52, *p* = 0.020, *ɳ*_*p*_^2^ = 0.266], showing that the children made fewer errors when solving trained simple problems than untrained simple problems (Fig. 2B). Further analysis revealed that the main effect of training comes from the post-test difference between trained and untrained simple problems [*t*(33.24) = 2.22, *p* = 0.033, *d* = 0.50], while there was no significant difference in the pre-test [*t*(33.24) = 1.37, *p* = 0.179, *d* = 0.31]. This observation suggests training-specific improvement as regards error rate in simple conditions. A significant interaction of training × grade [*F*(1,18) = 4.90, *p* = 0.040, *ɳ*_*p*_^2^ = 0.214] indicated a larger difference between trained and untrained conditions for older children [*r*(18) = 0.46, *p* = 0.040]. Other effects were not significant (see Supplementary Materials, Table S2).

#### Complex

The rmANCOVA of median RT for complex multiplication revealed a significant main effect of time [*F*(1,18) = 5.39, *p* = 0.032, *ɳ*_*p*_^2^ = 0.230], showing that the children became faster at complex multiplication after training (Fig. 2A). This observation suggests training improved performance in both trained [*t*(29.59) = 2.61, *p* = 0.014, *d* = 0.58] and untrained [*t*(29.59) = 2.43, *p* = 0.022, *d* = 0.54] complex problems over time. The significant main effect of training [*F*(1,18) = 4.59, *p* = 0.046, *ɳ*_*p*_^2^ = 0.203] indicated faster responses for trained complex problems than untrained complex problems. Further analysis revealed that the main effect of training comes from the post-test difference between trained and untrained complex problems [*t*(33.73) = 2.12, *p* = 0.041, *d* = 0.47], while the difference was marginally significant in the pre-test [*t*(33.24) = 1.84, *p* = 0.074, *d* = 0.41]. This observation suggests training-specific improvement as regards median RT in complex conditions. Furthermore, the significant main effect of grade [*F*(1,18) = 14.70, *p* = 0.001, *ɳ*_*p*_^2^ = 0.450] indicated that older children responded faster than younger children [*r*(18) = -0.67, *p* = 0.001].

The rmANCOVA of error rate revealed a significant main effect of time [*F*(1,18) = 4.39, *p* = 0.050, *ɳ*_*p*_^2^ = 0.196], showing that the children made fewer errors after training (Fig. 2B). Further analysis revealed a marginal effect of time in trained complex problems [*t*(27.47) = 1.83, *p* = 0.078, *d* = 0.41], but not in untrained complex problems [*t*(27.47) = 0.69, *p* = 0.499, *d* = 0.15]. The main effect of grade [*F*(1,18) = 11.60, *p* = 0.003, *ɳ*_*p*_^2^ = 0.391] indicated that older children made fewer errors than younger children [*r*(18) = − 0.63, *p* = 0.003]. Other effects were not significant (see Supplementary Materials, Table S2).

The correlation analysis between the RTs and error rates before training predicted the training effect (trained simple RT: *r*(18) = 0.70, *p* < 0.001; trained complex RT: *r*(18) = 0.91, *p* < 0.001; trained simple error rate: *r*(18) = 0.92, *p* < 0.001; trained complex error rate: *r*(18) = 0.88, *p* < 0.001). This finding suggests that children with poorer performance before training gained more from the 2 weeks of multiplication training.

### fNIRS

#### Simple

**Fig. 3:**
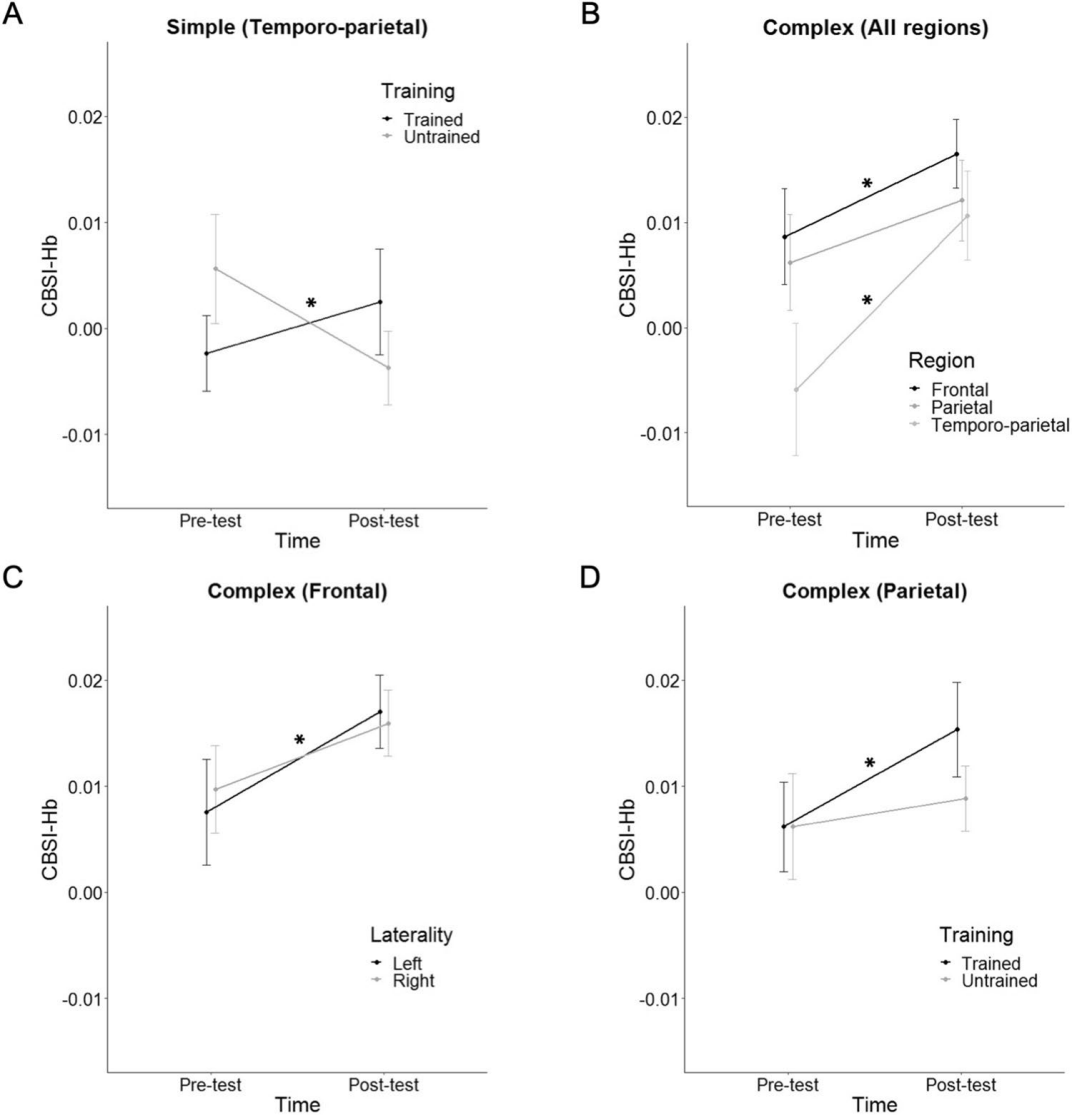
**A** Temporo-parietal activation increased for trained simple multiplication problems after 2 weeks of training and decreased for untrained simple multiplication problems. **B** Activation in frontal and temporo-parietal cortices, but not in the parietal cortex, significantly increased for trained and untrained complex multiplication problems after 2 weeks of training. **C** The activation increase in the left frontal cortex was significantly greater than in the right frontal cortex after 2 weeks of training. **D** Parietal activation significantly increased for trained complex multiplication problems after 2 weeks of training as compared to untrained complex multiplication problems. Error bars depict 1 *SE* of the mean. For simplicity, the values in the figure are not adjusted for the covariate. *p < 0.05

The rmANCOVA revealed a significant interaction of time × training in the temporo-parietal cortex [*F*(1,18) = 4.72, *p* = 0.043, *ɳ*_*p*_^2^ = 0.208], suggesting training-specific changes. Whereas temporo-parietal activation increased during trained simple multiplication solving after 2 weeks, this activation decreased during untrained simple multiplication solving (Fig. 3A). Other effects were not significant (see Supplementary Materials, Table S3).

#### Complex

In the frontal cortex, a significant main effect of time [*F*(1,18) = 6.46, *p* = 0.020, *ɳ*_*p*_^2^ = 0.264] and an interaction of time × grade [*F*(1,18) = 5.42, *p* = 0.032, *ɳ*_*p*_^2^ = 0.231] (Fig. 2B) showed increased activation after 2 weeks of training, particularly in younger children [*r*(18) = − 0.48, *p* = 0.032]. The significant interaction of time × hemisphere [*F*(1,18) = 6.37, *p* = 0.021, *ɳ*_*p*_^2^ = 0.261] and the interaction of time × hemisphere × grade [*F*(1,18) = 5.77, *p* = 0.027, *ɳ*_*p*_^2^ = 0.243] showed a greater increase in the left frontal activation than in the right frontal activation after 2 weeks of training (Fig. 3C), particularly in younger children.

In the parietal cortex, a significant main effect of hemisphere [*F*(1,18) = 5.14, *p* = 0.036, *ɳ*_*p*_^2^ = 0.222] and an interaction of hemisphere × grade [*F*(1,18) = 5.20, *p* = 0.035, *ɳ*_*p*_^2^ = 0.224] showed greater activation in the right than in the left parietal cortex, particularly in younger children [*r*(18) = 0.47, *p* = 0.035]. The significant interaction of time × training [*F*(1,18) = 5.63, *p* = 0.029, *ɳ*_*p*_^2^ = 0.238] and the interaction of time × training × grade [*F*(1,18) = 5.12, *p* = 0.036, *ɳ*_*p*_^2^ = 0.221] revealed training-specific changes. The parietal activation increased during trained complex multiplication after 2 weeks of training as compared to untrained complex multiplication (Fig. 3D), particularly in younger children.

In the temporo-parietal cortex, a significant main effect of time [*F*(1,18) = 6.21, *p* = 0.023, *ɳ*_*p*_^2^ = 0.256] showed increased activation after 2 weeks of training (Fig. 3B). Other effects were not significant (see Supplementary Materials, Table S4).

#### Global analysis

**Fig. 4:**
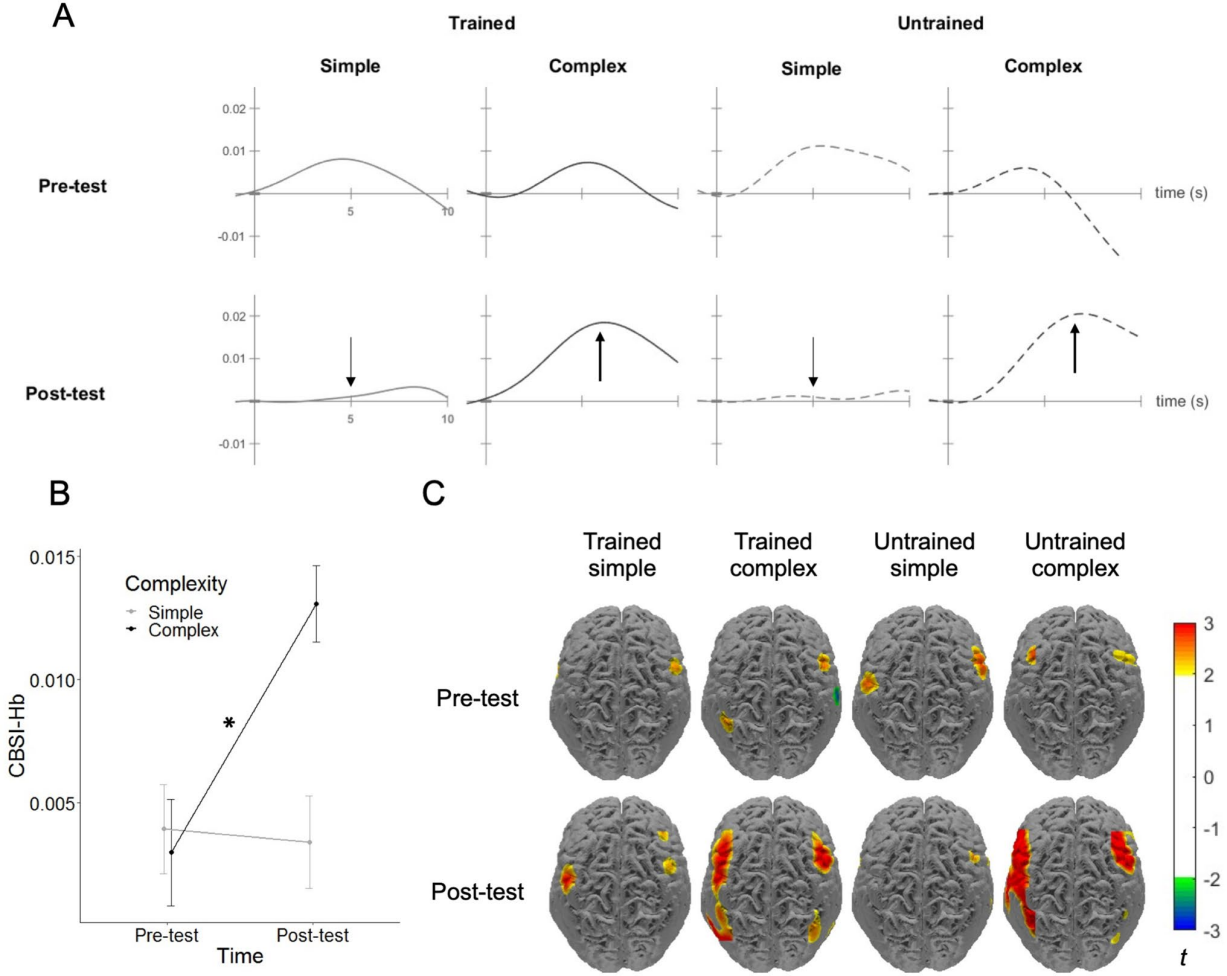
Difficulty-related modulation of neural activity: **A** The time course of global activation (i.e., mean activation of all 44 channels) for each condition in pre- and post-test. Simple and complex conditions are distinguishable in the post-test but not in the pre-test. **B** Global activation for simple (both trained and untrained) and complex (both trained and untrained) conditions in pre- and post-test. Error bars depict 1 *SE* of the mean. For simplicity, the values in the figure are not adjusted for the covariate. *p < 0.05. **C** The heat map of brain activation for each condition in pre- and post-test. Red and blue demonstrate increased and decreased activation, respectively

To test difficulty-related modulation of neural activity, the rmANCOVA on global activation revealed a significant interaction of time × complexity [*F*(1,18) = 4.84, *p* = 0.041, *ɳ*_*p*_^2^ = 0.212], showing increased activation only for complex multiplication after 2 weeks of training [*t*(35.96) = 2.06, *p* = 0.047, *d* = 0.46] (Fig. 4). Other effects were not significant (see Supplementary Materials, Table S5).

## Discussion

The present study aimed at uncovering the brain activation changes underlying arithmetic learning in children with MD. Children with MD underwent computer-based arithmetic training for 2 weeks. In a within-participant design, brain activation in children with MD was measured using fNIRS during simple and complex multiplication problem solving before and after the training, following up on our previous study in TD children (Soltanlou et al. 2018b).

### Neurobehavioral changes during simple calculation in children with MD

On the behavioral level, fewer errors were observed in the trained simple as compared to untrained simple problems in the post-test, showing that 2 weeks of training was effective (see also Supplementary Materials, Table S6 for transfer effects). On the neural level, 2 weeks of training led to training-specific changes: temporo-parietal activation increased for trained simple multiplication problems, but decreased for untrained simple multiplication problems. The left angular gyrus of the temporo-parietal cortex is a part of the arithmetic fact retrieval network and automatization (Dehaene et al. 2003; Klein et al. 2016). Our training probably led to the engagement of this network during simple multiplication problem solving, which indicates that the children used advanced strategies, such as rote retrieval strategy and automatized procedures, to a greater degree. This interpretation is in line with the reported strategy that showed higher fact retrieval strategy use after training as compared to before training measurement.

The increased temporo-parietal activation for trained simple multiplication supports previous studies showing reduced overall brain activation in children with MD as compared to TD children in a single-session measurement (Berteletti et al. 2014). However, it contradicts fronto-parietal activation decrease during simple addition problem solving that was reported by Iuculano et al. (2015). Several methodological differences between the two studies might explain this inconsistency. For example, our study was in 10–14 years old children who are more advanced in simple arithmetic skills than 7–10 years old children in their study who have just started learning those skills. Previous longitudinal (e.g., Artemenko et al. 2018) and cross-sectional (e.g., Rivera et al. 2005) have shown brain activation differences related to mathematical skills and advancements between younger and older children. Additionally, while our 2-week training focused on the repetition of similar multiplication problems (the most common way of learning multiplication at school), their 8-week one-to-one tutoring focused on conceptual knowledge acquisition such as counting strategies and speeded retrieval facts. In other words, while the aim of our study was developing retrieval strategy by repeating the same problems over and over, Iuculano et al. (2015) aimed to develop broader mathematical knowledge in children with MD. There are some other methodological differences between the two studies, such as number of training sessions (6 vs 16), arithmetic operation (multiplication vs addition), and paradigm (verbal production vs verification) that may partially explain the contradictory findings. However, because of a lack of knowledge in this field, more studies are needed to better understand the mechanisms of simple calculation training in individuals with MD.

While the left angular gyrus has frequently been associated with multiplication fact retrieval, the literature on the role of the right angular gyrus is less conclusive. Arsalidou and Taylor (2011) suggested that the right angular gyrus is involved in goal-directed salience representations and supports visuospatial attentional demands during mental arithmetic. We might conclude that children with MD became more fluent and automatized in their problem solving strategies only for the simple multiplication problems on which they received direct training but they could not transfer this fluency to untrained, new simple multiplication problems. This training-specific change derives from the interaction between measurement time and training, which shows an unexpectedly decreased temporo-parietal activation in untrained simple multiplication over time. Behavioral data indicate almost no improvement in untrained simple multiplication over time. Therefore, we might speculate that children with MD may have not yet established a problem solving strategy for untrained calculations that might lead to random brain activation patterns. Note that within our training procedure, we expected developing those strategies for trained problems, which we assume to lead to more consistent brain responses to the trained problems. Future studies using trial-by-trial strategy reports might help to better understand these brain activation changes.

### Neurobehavioral changes during complex calculation in children with MD

Children with MD improved in their behavioral performance in trained as compared to untrained complex arithmetic after training (see also Supplementary Materials, Table S6 for transfer effects). While they were faster in solving both trained and untrained complex problems after training, this improvement was significantly larger in trained complex problems. Children also made fewer errors only in trained but not untrained complex problems after training. On the neural level, training-related activation changes were exclusively observed in the parietal region and generalized to untrained complex multiplication in the frontal and temporo-parietal regions (Fig. 3B and D).

As one of the most interesting findings in the current study, 2 weeks of arithmetic training improved the numerical and spatial processes related to mental calculation. This was reflected by the training-related increase in parietal activation, particularly in younger children. The parietal cortex, i.e., the intraparietal sulcus and the superior parietal lobule, is involved in magnitude and visuospatial attentional processes during mental arithmetic (Dehaene et al. 2003). The increased engagement of these regions during trained complex problem solving but not untrained complex problems supports previous literature on the dysfunction of these regions in MD. For instance, Kucian et al. (2014) suggested an impaired or delayed development of axonal coherence and myelination in the fibers projecting to/from the parietal cortex in children with MD, which might point to a disconnection syndrome. We may conclude that 2 weeks of training partially resolved this disconnection syndrome and reduced the arithmetic deficit in children with MD. However, this conclusion is based on contrast analyses and the connectivity between the parietal cortices and other math-related brain regions needs to be investigated.

We also observed higher activation in the right than in the left parietal cortex, particularly in younger children. While the right intraparietal sulcus is engaged in non-symbolic number magnitude processing, particularly at early ages (Edwards et al. 2016; Hyde et al. 2010), the left intraparietal sulcus is deeply involved in symbolic number magnitude processing of the numbers at school ages (Vogel et al. 2015) in association with language-related areas on the left hemisphere. Supporting our current finding, the right parietal cortex is expected to be involved in the calculation to a greater degree in younger children than the left parietal cortex, according to a recent theoretical model of functional lateralization in the parietal cortex in arithmetic (Artemenko et al. 2020).

Two weeks of training improved the cognitive processes related to mental calculation, as frontal activation generally increased after training, particularly in the left hemisphere. This improvement was not training-specific and observed in both trained and untrained complex problem solving. Frontal activation within the fronto-parietal network is associated with domain-general cognitive processes during complex arithmetic (Klein et al. 2016). These frontal circuits are mainly involved in procedural strategies, which are the strategies usually applied by TD children for complex arithmetic (Arsalidou et al. 2018; Peters and De Smedt 2018). This frontal activation increase was higher for younger children, who exhibited poorer performance than older children before training. Therefore, it is likely that younger children gained more from training.

Two weeks of training improved the language-related and goal-directed processes related to mental calculation, as reflected by an activation increase in the temporo-parietal region, i.e., angular gyrus and middle temporal gyrus. Similar to the frontal activation increase, this improvement was not training-specific and observed for both trained and untrained complex problem solving. The left and right angular gyri are, respectively, engaged in language-related processes (Kesler et al. 2011) and goal-directed salience representations during mental arithmetic (Arsalidou and Taylor 2011). The increased involvement of these processes seems rational because, among the basic arithmetic operations, multiplication is the one that strongly relies on verbal repetitions and even complex multiplication usually is decomposed into simple facts that might be retrieved from memory. It seems that children with MD develop retrieval and automated procedural strategies, which lead to increased bilateral angular gyrus activation (Ashkenazi et al. 2013; Polspoel et al. 2017; Rykhlevskaia et al. 2009). It is worth noting that one of the most common procedures in complex calculation is to split the calculation into small steps which can be solved by retrieval or fast procedural processes (Soltanlou et al. 2018a, b). Therefore, retrieval processes are part of procedural strategies used during complex multiplication and increased bilateral angular gyrus activation within the temporo-parietal region is interpreted as a sign of training effectiveness. This interpretation is supported by higher retrieval strategies after 2 weeks of training as compared to before training (see Supplementary Material, Table S6).

### Difficulty-related modulation of neural activity

Two weeks of training led to the difficulty-related modulation of neural activity in children with MD (Ashkenazi et al. 2012): discriminable brain activation for simple versus complex multiplication problem solving was observed after training but not before training. This reflects the more extended brain activation pattern during complex than simple arithmetic, which is typically observed for TD children (e.g., Soltanlou et al. 2017a). Note that although this difficulty-related modulation effect is widespread, it is not simply a systemic effect because it comes from an interaction effect, where increased activation is only observed for complex multiplication and not simple multiplication, and the simple and complex problems were randomly presented during the current event-related design.

The development towards typical arithmetic processing by children with MD is supported by other training studies (Iuculano et al. 2015; Michels et al. 2018). Iuculano et al. (2015) found that brain activation in children with MD is significantly discriminable from TD peers only before, not after, training. In the same vein, Michels et al. (2018) reported discriminable functional connectivity between children with MD and TD children before, but not after, 5 weeks of number line training. The data from the current study indicate that 2 weeks of multiplication training activate the most relevant neural networks for arithmetic processing so that brain activation patterns for children with MD are like those of TD children from other studies.

### Limitations and future perspectives

We acknowledge our rather small sample size and broad age range in the current study and we are aware of the replicability crisis in psychology and neuroscience. However, we should mention that data collection in special populations is quite challenging and time-consuming. To overcome this barrier, and some others such as different MD definition criteria, comorbidity and heterogeneity, experimental design, and data-analytic methods (De Smedt et al. 2019; Kaufmann et al. 2013), we recommend multi-lab and multivariate approaches with a large *N* for future research.

In the absence of a direct control group within the same statistical models, the current study followed our previous multiplication training study in TD children (Soltanlou et al. 2018b) and results were compared between these studies with almost the same procedure. The specific findings for trained but not untrained arithmetic after 2 weeks of training in children with MD prove training specificity rather than a regression-to-the-mean effect in our data, which might be a concern in the absence of a direct control group.

Our training method reflected the common way of teaching multiplication at school (repetition of multiplication problems), which may not necessarily improve mathematical understanding, but led to an improvement in both response time and accuracy. While the response time might be related to retrieval processes, the accuracy cannot be directly interpreted as an improved retrieval strategy. On the one hand, improved retrieval strategies might increase children’s self-confidence and lead to lower missing responses that were considered as errors in this study. It is also possible that the retrieval strategy is more accurate than a procedural strategy, particularly in children, who have problems with domain-specific or domain-general processes in the procedural strategy. On the other hand, even improvement in response time is not solely related to fact retrieval strategies, but automatic mapping processes (De Visscher et al. 2015) and compact procedural strategies (Soltanlou et al. 2018a, b), which are not necessarily part of fact retrieval strategies. These arguments are supported by the training-specific changes in the IPS and also activation increase in the prefrontal cortex, which are, respectively, related to the manipulation of symbolic numbers and supportive cognitive processes during mental calculation rather than retrieval processes. However, some of our findings such as training-related activation increase in AG during simple multiplication problem solving might be also observed after training of a non-numerical task. For instance, Grabner et al. (2009) showed an unspecific activation increase in the left AG during both multiplication and figural-spatial problems solving. Since we did not include any other mathematical task to test for the general mathematical understanding of children, we can neither exclude better retrieval strategies nor more compact procedural strategies as explanations of our findings. In fact, the available literature suggests the interpretation that training improvement may not be either better fact retrieval or more compact procedural strategies, but could be well due to both and depend on individual and problems to be solved.

As a methodological limitation, fNIRS with its restricted depth resolution only allows for the measurement of about 1–1.5 cm of the cortex (corresponding to 3 cm from the scalp) in adults (Haeussinger et al. 2011; Schroeter et al. 2006). Therefore, we were not able to record activation changes in subcortical regions such as the hippocampus that are involved in mental arithmetic (e.g., Klein et al. 2016).

## Conclusion

This study followed our recent multiplication training in TD children (Soltanlou et al. 2018b) and investigated arithmetic learning in children with MD. A two-week training by an interactive learning platform improved behavioral arithmetic performance in both TD children and children with MD. Surprisingly, opposite brain activation changes were observed in TD children (Soltanlou et al. 2018b) and in children with MD (current study): While 2 weeks of multiplication training led to brain activation decrease in the fronto-parietal network of mental calculation in TD children, the same training led to brain activation increase in that network in children with MD. However, these differences between the current findings and the findings in TD children (Soltanlou et al. 2018a, b) need to be interpreted cautiously as there was no direct statistical comparison between the two studies. We conclude that: First the neural mechanisms of arithmetic learning differ between TD children and children with MD, Second, while calculation improvement relates to more automatized and less effortful processes in TD children, this improvement relates to more and functionally better usage of cognitive processes in children with MD. Third, the application of neuroimaging in the educational context provides valuable insights about the underlying mechanisms of mathematics learning (Dresler et al. 2018). Namely, behavioral improvements may not necessarily rely on similar neurocognitive changes in children with and without mathematics problems. Fourth, educational interventions should develop based on the direct findings in individuals with MD rather than generalizations of findings in typical populations. In other words, interventions that are developed based on observations in TD children may not be the best ways to improve mathematical skills in children with MD and more direct studies in children with MD may provide a more beneficial guidance.

## Supplementary Information

Below is the link to the electronic supplementary material.

Supplementary file1 (DOC 189 KB)

## Data Availability

The data that support the findings of this study are available on request from the corresponding author. The data are not publicly available due to privacy or ethical restrictions.

## References

[CR1] Arsalidou M, Taylor MJ (2011). Is 2+2=4? Meta-analyses of brain areas needed for numbers and calculations. Neuroimage.

[CR2] Arsalidou M, Pawliw-Levac M, Sadeghi M, Pascual-Leone J (2018). Brain areas associated with numbers and calculations in children: Meta-analyses of fMRI studies. Dev Cogn Neurosci.

[CR3] Artemenko C, Soltanlou M, Ehlis A-C, Nuerk H-C, Dresler T (2018). The neural correlates of mental arithmetic in adolescents: a longitudinal fNIRS study. Behav Brain Funct.

[CR4] Artemenko C, Sitnikova MA, Soltanlou M, Dresler T, Nuerk H-C (2020). Functional lateralization of arithmetic processing in the intraparietal sulcus is associated with handedness. Sci Rep.

[CR5] Ashkenazi S, Rosenberg-Lee M, Tenison C, Menon V (2012). Weak task-related modulation and stimulus representations during arithmetic problem solving in children with developmental dyscalculia. Dev Cogn Neurosci.

[CR6] Ashkenazi S, Black JM, Abrams DA, Hoeft F, Menon V (2013). Neurobiological underpinnings of math and reading learning disabilities. J Learn Disabil.

[CR7] Berteletti I, Prado J, Booth JR (2014). Children with mathematical learning disability fail in recruiting verbal and numerical brain regions when solving simple multiplication problems. Cortex.

[CR8] Brigadoi S, Ceccherini L, Cutini S, Scarpa F, Scatturin P, Selb J (2014). Motion artifacts in functional near-infrared spectroscopy: a comparison of motion correction techniques applied to real cognitive data. Neuroimage.

[CR9] Corsi PM (1973) Human memory and the medial temporal region of the brain*.* ProQuest Information & Learning

[CR10] Cui X, Bray S, Reiss AL (2010). Functional near infrared spectroscopy (NIRS) signal improvement based on negative correlation between oxygenated and deoxygenated hemoglobin dynamics. Neuroimage.

[CR11] Davis N, Cannistraci CJ, Rogers BP, Gatenby JC, Fuchs LS, Anderson AW, Gore JC (2009). Aberrant functional activation in school age children at-risk for mathematical disability: a functional imaging study of simple arithmetic skill. Neuropsychologia.

[CR12] De Smedt B, Peters L, Ghesquière P, Fritz A, Haase VG, Räsänen P (2019). Neurobiological origins of mathematical learning disabilities or dyscalculia: a review of brain imaging data. International handbook of mathematical learning difficulties.

[CR13] De Visscher A, Berens SC, Keidel JL, Noël MP, Bird CM (2015). The interference effect in arithmetic fact solving: an fMRI study. Neuroimage.

[CR14] Dehaene S, Piazza M, Pinel P, Cohen L (2003). Three parietal circuits for number processing. Cogn Neuropsychol.

[CR15] Dresler T, Bugden S, Gouet C, Lallier M, Oliveira DG, Pinheiro-Chagas P (2018). A translational framework of educational neuroscience in learning disorders. Front Integr Neurosci.

[CR16] Edwards LA, Wagner JB, Simon CE, Hyde DC (2016). Functional brain organization for number processing in pre-verbal infants. Dev Sci.

[CR17] Ehlert A, Schroeders U, Fritz-Stratmann A (2012). Criticism of the discrepancy criterion in the diagnosis of dyslexia and dyscalculia. Lernen und Lernstörungen.

[CR18] Fishburn FA, Ludlum RS, Vaidya CJ, Medvedev AV (2019). Temporal derivative distribution repair (TDDR): a motion correction method for fNIRS. Neuroimage.

[CR19] Grabner RH, Ischebeck A, Reishofer G, Koschutnig K, Delazer M, Ebner F, Neuper C (2009). Fact learning in complex arithmetic and figural-spatial tasks: the role of the angular gyrus and its relation to mathematical competence. Hum Brain Mapp.

[CR20] Gross J, Hudson C, Price D (2009). The long term costs of numeracy difficulties.

[CR21] Haeussinger FB, Heinzel S, Hahn T, Schecklmann M, Ehlis A-C, Fallgatter AJ (2011). Simulation of near-infrared light absorption considering individual head and prefrontal cortex anatomy: implications for optical neuroimaging. PLoS ONE.

[CR22] Haeussinger FB, Dresler T, Heinzel S, Schecklmann M, Fallgatter AJ, Ehlis AC (2014). Reconstructing functional near-infrared spectroscopy (fNIRS) signals impaired by extra-cranial confounds: an easy-to-use filter method. Neuroimage.

[CR23] Hyde DC, Boas DA, Blair C, Carey S (2010). Near-infrared spectroscopy shows right parietal specialization for number in pre-verbal infants. Neuroimage.

[CR24] Iuculano T, Rosenberg-Lee M, Richardson J, Tenison C, Fuchs L, Supekar K, Menon V (2015). Cognitive tutoring induces widespread neuroplasticity and remediates brain function in children with mathematical learning disabilities. Nat Commun.

[CR25] jamovi project (2019) jamovi (Version 1). [Computer Software]. Retrieved from https://www.jamovi.org

[CR26] Jolles D, Ashkenazi S, Kochalka J, Evans T, Richardson J, Rosenberg-Lee M (2016). Parietal hyper-connectivity, aberrant brain organization, and circuit-based biomarkers in children with mathematical disabilities. Dev Sci.

[CR27] Kaufmann L, Wood G, Rubinsten O, Henik A (2011). Meta-analyses of developmental fMRI studies investigating typical and atypical trajectories of number processing and calculation. Dev Neuropsychol.

[CR28] Kaufmann L, Mazzocco MM, Dowker A, von Aster M, Gobel SM, Grabner RH (2013). Dyscalculia from a developmental and differential perspective. Front Psychol.

[CR29] Kesler SR, Sheau K, Koovakkattu D, Reiss AL (2011). Changes in frontal-parietal activation and math skills performance following adaptive number sense training: preliminary results from a pilot study. Neuropsychol Rehabil.

[CR30] Klein E, Suchan J, Moeller K, Karnath HO, Knops A, Wood G (2016). Considering structural connectivity in the triple code model of numerical cognition: differential connectivity for magnitude processing and arithmetic facts. Brain Struct Funct.

[CR31] Kucian K, von Aster M (2015). Developmental dyscalculia. Eur J Pediatr.

[CR32] Kucian K, Loenneker T, Dietrich T, Dosch M, Martin E, von Aster M (2006). Impaired neural networks for approximate calculation in dyscalculic children: a functional MRI study. Behav Brain Funct.

[CR33] Kucian K, Grond U, Rotzer S, Henzi B, Schönmann C, Plangger F (2011). Mental number line training in children with developmental dyscalculia. Neuroimage.

[CR34] Kucian K, Ashkenazi SS, Hanggi J, Rotzer S, Jancke L, Martin E, von Aster M (2014). Developmental dyscalculia: a dysconnection syndrome?. Brain Struct Funct.

[CR35] McCaskey U, von Aster M, Maurer U, Martin E, O'Gorman Tuura R, Kucian K (2018). Longitudinal brain development of numerical skills in typically developing children and children with developmental dyscalculia. Front Hum Neurosci.

[CR100] McCaskey U, von Aster M, O’Gorman R, Kucian K (2020). Persistent differences in brain structure in developmental dyscalculia: a longitudinal morphometry study. Front Hum Neurosci.

[CR36] Michels L, O'Gorman R, Kucian K (2018). Functional hyperconnectivity vanishes in children with developmental dyscalculia after numerical intervention. Dev Cogn Neurosci.

[CR37] Moser Opitz E, Ramseier E, Reusser L, Haselhorn M, Heinzel A, Schneider W, Trautwein U (2010). Basisdiagnostik Mathematik für die Klassen 4–8 (BASIS-Math 4–8). Jahrbuch Der Pädagogisch-Psychologischen Diagnostik. Tests Und Trends. Neue Folge.

[CR38] Petermann F, Petermann U, Wechsler D (2007) Hamburg-Wechsler-Intelligenztest für Kinder-IV: HAWIK-IV: Huber

[CR39] Peters L, De Smedt B (2018). Arithmetic in the developing brain: a review of brain imaging studies. Dev Cogn Neurosci.

[CR40] Peters L, Bulthe J, Daniels N, Op de Beeck H, De Smedt B (2018). Dyscalculia and dyslexia: different behavioral, yet similar brain activity profiles during arithmetic. NeuroImage: Clinical.

[CR41] Polspoel B, Peters L, Vandermosten M, De Smedt B (2017). Strategy over operation: neural activation in subtraction and multiplication during fact retrieval and procedural strategy use in children. Hum Brain Mapp.

[CR42] R Core Team (2018) R: A Language and Environment for Statistical Computing. Vienna, Austria. Retrieved from https://www.R-project.org/

[CR43] Ranpura A, Isaacs E, Edmonds C, Rogers M, Lanigan J, Singhal A (2013). Developmental trajectories of grey and white matter in dyscalculia. Trends Neurosci Educ.

[CR44] Rivera SM, Reiss AL, Eckert MA, Menon V (2005). Developmental changes in mental arithmetic: evidence for increased functional specialization in the left inferior parietal cortex. Cereb Cortex.

[CR45] Rorden C, Brett M (2000). Stereotaxic display of brain lesions. Behav Neurol.

[CR46] Rosenberg-Lee M, Ashkenazi S, Chen T, Young CB, Geary DC, Menon V (2015). Brain hyper-connectivity and operation-specific deficits during arithmetic problem solving in children with developmental dyscalculia. Dev Sci.

[CR47] Rotzer S, Kucian K, Martin E, von Aster M, Klaver P, Loenneker T (2008). Optimized voxel-based morphometry in children with developmental dyscalculia. Neuroimage.

[CR48] Rykhlevskaia E, Uddin LQ, Kondos L, Menon V (2009). Neuroanatomical correlates of developmental dyscalculia: combined evidence from morphometry and tractography. Front Hum Neurosci.

[CR49] Sasai S, Homae F, Watanabe H, Taga G (2011). Frequency-specific functional connectivity in the brain during resting state revealed by NIRS. Neuroimage.

[CR50] Scholkmann F, Kleiser S, Metz AJ, Zimmermann R, Pavia JM, Wolf U, Wolf M (2014). A review on continuous wave functional near-infrared spectroscopy and imaging instrumentation and methodology. Neuroimage.

[CR51] Schroeter ML, Kupka T, Mildner T, Uludağ K, von Cramon DY (2006). Investigating the post-stimulus undershoot of the BOLD signal—a simultaneous fMRI and fNIRS study. Neuroimage.

[CR52] Schwartz F, Epinat-Duclos J, Leone J, Poisson A, Prado J (2018). Impaired neural processing of transitive relations in children with math learning difficulty. NeuroImage: Clinical.

[CR53] Simos PG, Kanatsouli K, Fletcher JM, Sarkari S, Juranek J, Cirino P (2008). Aberrant spatiotemporal activation profiles associated with math difficulties in children: a magnetic source imaging study. Neuropsychology.

[CR54] Singh AK, Okamoto M, Dan H, Jurcak V, Dan I (2005). Spatial registration of multichannel multi-subject fNIRS data to MNI space without MRI. Neuroimage.

[CR55] Soltanlou M, Artemenko C, Dresler T, Haeussinger FB, Fallgatter AJ, Ehlis AC, Nuerk HC (2017). Increased arithmetic complexity is associated with domain-general but not domain-specific magnitude processing in children: a simultaneous fNIRS-EEG study. Cogn Affect Behav Neurosci.

[CR56] Soltanlou M, Jung S, Roesch S, Ninaus M, Brandelik K, Heller J et al. (2017b) Behavioral and neurocognitive evaluation of a web-platform for game-based learning of orthography and numeracy. In: Informational Environments. Springer, pp 149–176

[CR57] Soltanlou M, Sitnikova MA, Nuerk H-C, Dresler T (2018). Applications of functional near-Infrared spectroscopy (fNIRS) in studying cognitive development: the case of mathematics and language. Front Psychol.

[CR58] Soltanlou M, Artemenko C, Ehlis A-C, Huber S, Fallgatter AJ, Dresler T, Nuerk H-C (2018). Reduction but no shift in brain activation after arithmetic learning in children: a simultaneous fNIRS-EEG study. Sci Rep.

[CR101] Soltanlou M, Artemenko C (2020). Using light to understand how the brain works in the classroom. Front Young Mind.

[CR59] Tong Y, Frederick BD (2010). Time lag dependent multimodal processing of concurrent fMRI and near-infrared spectroscopy (NIRS) data suggests a global circulatory origin for low-frequency oscillation signals in human brain. Neuroimage.

[CR60] Tsuzuki D, Jurcak V, Singh AK, Okamoto M, Watanabe E, Dan I (2007). Virtual spatial registration of stand-alone fNIRS data to MNI space. Neuroimage.

[CR61] Tzourio-Mazoyer N, Landeau B, Papathanassiou D, Crivello F, Etard O, Delcroix N (2002). Automated anatomical labeling of activations in SPM using a macroscopic anatomical parcellation of the MNI MRI single-subject brain. Neuroimage.

[CR62] Vogel SE, Goffin C, Ansari D (2015). Developmental specialization of the left parietal cortex for the semantic representation of Arabic numerals: an fMR-adaptation study. Dev Cogn Neurosci.

[CR63] Wimmer H, Mayringer H (2014) SLS 2–9: Salzburger Lese-Screening für die Schulstufen 2–9. Huber, Hogrefe, Göttingen

[CR64] Zamarian L, Ischebeck A, Delazer M (2009). Neuroscience of learning arithmetic—evidence from brain imaging studies. Neurosci Biobehav Rev.

