# Supplementary material for: Training causes activation increase in temporo-parietal and parietal regions in children with mathematical disabilities
Source: Brain Struct Funct. 2022 Mar 7;227(5):1757–71. doi: 10.1007/s00429-022-02470-5 (PMC9098620; doi:10.1007/s00429-022-02470-5)
Supplement: Supplementary file 1 — Supplementary file1 (DOC 189 KB) [file 429_2022_2470_MOESM1_ESM.doc]

**Supplementary Materials for**

**Training causes activation increase in temporo-parietal and parietal regions in children with mathematical disabilities**

Mojtaba Soltanlou, Thomas Dresler, Christina Artemenko, David Rosenbaum, Ann-Christine Ehlis, Hans-Christoph Nuerk

**Correlation between demographic information and neuropsychological tests**

Pearson correlation was conducted between demographic information and neuropsychological data (Table S1). The correlation analysis revealed that children in higher grades, who are older, had better non-verbal IQ and mathematics knowledge. Children who had better verbal IQ, had better verbal working memory as well. Children who had better non-verbal IQ, had better visuospatial short-term memory and mathematics knowledge.

Table S1: Pearson correlations between demographic information and neuropsychological data.

|  | **1** | **2** | **3** | **4** | **5** | **6** | **7** | **8** | **9** |
| --- | --- | --- | --- | --- | --- | --- | --- | --- | --- |
| **1. Age** | — |  |  |  |  |  |  |  |  |
| **2. Grade** | **0.83** | — |  |  |  |  |  |  |  |
| **3. Verbal IQ** | -0.07 | 0.06 | — |  |  |  |  |  |  |
| **4. Non-verbal IQ** | 0.23 | **0.48** | 0.44 | — |  |  |  |  |  |
| **5. Verbal short-term memory** | 0.17 | 0.09 | -0.14 | -0.17 | — |  |  |  |  |
| **6. Verbal working memory** | 0.01 | 0.15 | **0.45** | 0.44 | -0.40 | — |  |  |  |
| **7. Visuospatial short-term memory** | 0.20 | 0.18 | -0.08 | **0.54** | -0.31 | 0.16 | — |  |  |
| **8. Visuospatial working memory** | 0.03 | 0.24 | -0.36 | 0.07 | -0.04 | 0.13 | 0.16 | — |  |
| **9. Reading** | -0.22 | 0.03 | 0.14 | 0.21 | 0.01 | 0.11 | -0.05 | -0.17 | — |
| **10. Mathematics knowledge** | 0.39 | **0.54** | 0.41 | **0.55** | 0.00 | 0.44 | 0.16 | 0.19 | 0.22 |

Note: *N* = 20 (but 19 for the letter span - backward, and 18 for the reading test); uncorrected *p* < .05, two-tailed; significant results are shown in bold.

**Report of rmANCOVAs on behavioral and fNIRS data**

Table S2: Behavioral results of rmANCOVAs of median RT and error rate for simple and complex multiplication.

|  | ***Simple*** | | | ***Complex*** | | |
| --- | --- | --- | --- | --- | --- | --- |
|  | *F* | *p* | *ɳ_p_^2^* | *F* | *p* | *ɳ_p_^2^* |
| **Median RT** |  |  |  |  |  |  |
| **Time** | 0.01 | .995 | .001 | 5.39 | .**032** | .230 |
| **Time × Grade** | 0.14 | .712 | .008 | 3.47 | .079 | .162 |
| **Training** | 2.63 | .122 | .128 | 4.59 | **.046** | .203 |
| **Training × Grade** | 1.64 | .216 | .084 | 2.65 | .121 | .128 |
| **Time × Training** | 0.36 | .559 | .019 | 0.91 | .352 | .048 |
| **Time × Training × Grade** | 1.02 | .326 | .054 | 1.00 | .331 | .052 |
| **Grade** | 3.29 | .086 | .155 | 14.70 | .**001** | .450 |
| **Error rate** |  |  |  |  |  |  |
| **Time** | 1.02 | .327 | .053 | 4.39 | **.050** | .196 |
| **Time × Grade** | 1.64 | .217 | .084 | 3.56 | .075 | .165 |
| **Training** | 6.52 | **.020** | .266 | 0.19 | .666 | .011 |
| **Training × Grade** | 4.90 | **.040** | .214 | 0.07 | .800 | .004 |
| **Time × Training** | 2.50 | .132 | .122 | 0.21 | .650 | .012 |
| **Time × Training × Grade** | 2.20 | .155 | .109 | 0.07 | .795 | .004 |
| **Grade** | 2.35 | .142 | .116 | 11.60 | **.003** | .391 |

Note: *N* = 20; *df* = (1,18); significant results are shown in bold.

Table S3: fNIRS results of rmANCOVAs for simple multiplication.

|  | ***Frontal*** | | | ***Parietal*** | | | ***Temporo-parietal*** | | |
| --- | --- | --- | --- | --- | --- | --- | --- | --- | --- |
|  | *F* | *p* | *ɳ_p_^2^* | *F* | *p* | *ɳ_p_^2^* | *F* | *p* | *ɳ_p_^2^* |
| **Time** | 1.27 | .275 | .066 | 1.88 | .187 | .095 | 1.90 | .185 | .095 |
| **Time × Grade** | 1.27 | .275 | .066 | 2.02 | .172 | .101 | 1.76 | .201 | .089 |
| **Training** | 2.04 | .170 | .102 | 1.69 | .210 | .086 | 0.73 | .404 | .039 |
| **Training × Grade** | 2.21 | .155 | .109 | 1.96 | .178 | .098 | 0.71 | .411 | .038 |
| **Hemisphere** | 0.28 | .602 | .015 | 0.07 | .789 | .004 | 2.81 | .111 | .135 |
| **Hemisphere × Grade** | 0.49 | .495 | .026 | 0.22 | .648 | .012 | 2.40 | .139 | .118 |
| **Time × Training** | 0.14 | .715 | .008 | 1.79 | .197 | .091 | 4.72 | .**043** | .208 |
| **Time × Training × Grade** | 0.09 | .770 | .005 | 1.62 | .220 | .082 | 3.95 | .062 | .180 |
| **Time × Hemisphere** | 0.97 | .338 | .051 | 0.13 | .720 | .007 | 1.64 | .216 | .084 |
| **Time × Hemisphere × Grade** | 0.42 | .527 | .023 | 0.08 | .778 | .005 | 1.72 | .206 | .087 |
| **Training × Hemisphere** | 0.21 | .654 | .011 | 0.07 | .794 | .004 | 1.51 | .235 | .077 |
| **Training × Hemisphere × Grade** | 0.51 | .484 | .028 | 0.02 | .901 | .001 | 1.26 | .276 | .065 |
| **Time × Training × Hemisphere** | 0.04 | .850 | .002 | 0.21 | .649 | .012 | 0.95 | .342 | .050 |
| **Time × Training × Hemisphere × Grade** | 0.15 | .704 | .008 | 0.48 | .497 | .026 | 0.69 | .416 | .037 |
| **Grade** | 0.36 | .557 | .020 | 1.41 | .250 | .073 | 1.54 | .231 | .079 |

Note: *N* = 20; *df* = (1,18); significant results are shown in bold.

Table S4: fNIRS results of rmANCOVAs for complex multiplication.

|  | ***Frontal*** | | | ***Parietal*** | | | ***Temporo-parietal*** | | |
| --- | --- | --- | --- | --- | --- | --- | --- | --- | --- |
|  | *F* | *p* | *ɳ_p_^2^* | *F* | *p* | *ɳ_p_^2^* | *F* | *p* | *ɳ_p_^2^* |
| **Time** | 6.46 | **.020** | .264 | 1.31 | .268 | .068 | 6.21 | **.023** | .256 |
| **Time × Grade** | 5.42 | **.032** | .231 | 0.92 | .350 | .049 | 4.35 | .052 | .194 |
| **Training** | 0.32 | .577 | .018 | 0.06 | .814 | .003 | 0.48 | .499 | .026 |
| **Training × Grade** | 0.29 | .596 | .016 | 0.12 | .731 | .007 | 0.83 | .374 | .044 |
| **Hemisphere** | 1.79 | .197 | .091 | 5.14 | **.036** | .222 | 1.17 | .293 | .061 |
| **Hemisphere × Grade** | 1.73 | .205 | .088 | 5.20 | **.035** | .224 | 0.72 | .406 | .039 |
| **Time × Training** | 1.48 | .239 | .076 | 5.63 | **.029** | .238 | 0.87 | .363 | .046 |
| **Time × Training × Grade** | 1.75 | .202 | .089 | 5.12 | **.036** | .221 | 0.72 | .406 | .039 |
| **Time × Hemisphere** | 6.37 | **.021** | .261 | 0.63 | .439 | .034 | 0.01 | .928 | .000 |
| **Time × Hemisphere × Grade** | 5.77 | **.027** | .243 | 0.70 | .424 | .036 | 0.04 | .843 | .002 |
| **Training × Hemisphere** | 0.06 | .815 | .003 | 0.01 | .978 | .001 | 0.56 | .464 | .030 |
| **Training × Hemisphere × Grade** | 0.05 | .822 | .003 | 0.07 | .791 | .004 | 0.79 | .385 | .042 |
| **Time × Training × Hemisphere** | 0.84 | .371 | .045 | 3.38 | .082 | .158 | 1.51 | .235 | .078 |
| **Time × Training × Hemisphere × Grade** | 0.81 | .380 | .043 | 3.31 | .085 | .155 | 2.16 | .159 | .107 |
| **Grade** | 0.70 | .415 | .037 | 0.23 | .641 | .012 | 0.98 | .336 | .051 |

Note: *N* = 20; *df* = (1,18); significant results are shown in bold.

Table S5: fNIRS results of the rmANCOVA for global analysis.

|  | ***F*** | ***p*** | ***ɳ_p_^2^*** |
| --- | --- | --- | --- |
| **Time** | 0.52 | .479 | .028 |
| **Time × Grade** | 0.29 | .599 | .016 |
| **Complexity** | 2.21 | .155 | .109 |
| **Complexity × Grade** | 2.53 | .129 | .123 |
| **Time × Complexity** | 4.84 | **.041** | .212 |
| **Time × Complexity × Grade** | 3.78 | .068 | .173 |
| **Grade** | 0.01 | .930 | .001 |

Note: *N* = 20; *df* = (1,18); significant results are shown in bold.

**Behavioral training effects**

Similar to Kucian et al. (2011), some additional tests were administered before and after training. The tests included a general arithmetic ability test, a strategy questionnaire (Soltanlou et al., 2018), and information about self-concept, attitude towards math, and concerns about math (Soltanlou et al., 2019) (Table S6). To uncover the generalization and transfer effect of the two-week multiplication training in the above-mentioned tasks, paired *t*-tests (post-test versus pre-test) were conducted (Table S6).

The arithmetic ability test contained four basic operations (addition, subtraction, multiplication, and division) with two difficulty levels (simple and complex). Children were asked to solve as many problems as they could in a given time. They had 45 s for each list of simple problems and 60 s for each list of complex problems. Children showed better arithmetic performance (i.e., more correctly solved problems) after two weeks of training. This improved performance was significant for simple multiplication (near transfer), and complex subtraction and division (far transfer; Table S6).

The strategy questionnaire consisted of two multiplication problems from each of the four experimental conditions (simple/complex and trained/untrained) and was provided in four matched versions without a time limit. The children reported how they came to the solution after each problem. According to the child’s report, experimenters categorized each strategy as retrieval, procedural, or other (Soltanlou et al., 2018). Interestingly, children with DD reported higher utilization of the retrieval strategy after training (Table S6).

As it has been explained by Soltanlou et al. (2019), self-concept, attitude towards math, and concerns about math are part of the German translation of the math anxiety questionnaire (MAQ, Krinzinger et al., 2007; the original English version by Thomas & Dowker, 2000), which has an internal consistency (Cronbach’s alpha) of 0.83–0.91 for the whole questionnaire for different age groups. Each subscale contains five items describing different math-related topics. Each item needs to be rated on a five-point Likert scale (0 to 4) with a maximum score of 20 on each subscale. Thereby, higher values indicate a higher self-concept, a more positive attitude towards math, and less concern about math, respectively. While the self-concept in math and the attitude towards math subscales demonstrate general math-related attitudes, the concerns subscale about math indicates math anxiety (Krinzinger, Kaufmann, & Willmes, 2009). Children with DD reported a higher self-concept in math and lower math anxiety after two weeks of training (Table S6).

Table S6: Pre- and post-test comparisons for arithmetic ability (addition, subtraction, multiplication, division), strategy use, and math anxiety.

|  |  | **Pre-test** | | **Post-test** | | **Range** | ***t*(19)** | ***p*** | ***d*** |
| --- | --- | --- | --- | --- | --- | --- | --- | --- | --- |
|  |  | ***Mean*** | ***SD*** | ***Mean*** | ***SD*** |  |  |  |  |
| **Addition** | Simple | 10.40 | 2.41 | 10.20 | 2.14 | 0-18 | -0.48 | .680 | -0.11 |
|  | Complex | 8.20 | 2.31 | 7.95 | 2.04 | 0-18 | -0.68 | .747 | -0.15 |
| **Subtraction** | Simple | 8.30 | 2.23 | 8.45 | 2.19 | 0-15 | 0.30 | .383 | 0.07 |
|  | Complex | 4.60 | 2.14 | 5.50 | 2.06 | 0-15 | 2.10 | **.025** | 0.47 |
| **Multiplication** | Simple | 7.65 | 4.04 | 8.90 | 3.91 | 0-20 | 2.16 | **.022** | 0.48 |
|  | Complex | 2.45 | 1.73 | 2.80 | 1.85 | 0-13 | 1.05 | .154 | 0.23 |
| **Division** | Simple | 8.65 | 5.15 | 9.15 | 4.68 | 0-28 | 0.64 | .266 | 0.14 |
|  | Complex | 2.00 | 1.65 | 2.75 | 1.83 | 0-14 | 2.68 | **.007** | 0.60 |
| **Strategy** | Retrieval | 2.40 | 1.27 | 3.35 | 1.35 | 0-8 | 2.76 | **.006** | 0.62 |
|  | Procedural | 4.45 | 1.90 | 4.10 | 1.33 | 0-8 | -0.73 | .239 | -0.16 |
| **Math anxiety** | Self-concept | 11.45 | 3.52 | 12.30 | 3.48 | 0-20 | 2.74 | **.006** | 0.61 |
|  | Attitude | 11.15 | 3.17 | 11.75 | 3.09 | 0-20 | 1.50 | .075 | 0.34 |
|  | Anxiety | 6.10 | 3.06 | 7.00 | 2.88 | 0-20 | 1.81 | **.043** | 0.41 |

Note: *N* = 20; uncorrected *p* < .05, one-tailed; significant results are shown in bold.

**Experimental trials**

The stimulus set consisted of 32 multiplication problems with 8 problems in each of the 4 conditions (Soltanlou et al., 2018).

Table S7: Multiplication stimulus sets per condition.

| **Trained simple** | **Trained complex** | **Untrained simple** | **Untrained complex** |
| --- | --- | --- | --- |
| 3 × 4 | 13 × 4 | 6 × 2 | 18 × 3 |
| 5 × 3 | 3 × 19 | 7 × 2 | 6 × 12 |
| 2 × 8 | 5 × 13 | 3 × 7 | 4 × 19 |
| 6 × 3 | 18 × 4 | 4 × 6 | 7 × 12 |
| 3 × 9 | 6 × 13 | 8 × 3 | 14 × 6 |
| 7 × 4 | 15 × 6 | 7 × 5 | 17 × 5 |
| 5 × 6 | 12 × 8 | 4 × 9 | 5 × 18 |
| 8 × 4 | 7 × 14 | 5 × 8 | 13 × 7 |

**References**

Krinzinger, H., Kaufmann, L., & Willmes, K. (2009). Math anxiety and math ability in early primary school years. *Journal of psychoeducational assessment, 27*(3), 206-225.

Krinzinger, H., Kaufmann, L., Dowker, A., Thomas, G., Graf, M., Nuerk, H.-C., & Willmes, K. (2007). Deutschsprachige Version des Fragebogens für Rechenangst (FRA) für 6-bis 9-jährige Kinder. *Zeitschrift für Kinder-und Jugendpsychiatrie und Psychotherapie, 35*(5), 341-351.

Kucian, K., Grond, U., Rotzer, S., Henzi, B., Schönmann, C., Plangger, F., . . . von Aster, M. (2011). Mental number line training in children with developmental dyscalculia. *Neuroimage, 57*(3), 782-795.

Soltanlou, M., Artemenko, C., Dresler, T., Fallgatter, A. J., Ehlis, A. C., & Nuerk, H. C. (2019). Math Anxiety in Combination With Low Visuospatial Memory Impairs Math Learning in Children. *Frontiers in psychology, 10*, 89.

Soltanlou, M., Artemenko, C., Ehlis, A.-C., Huber, S., Fallgatter, A. J., Dresler, T., & Nuerk, H.-C. (2018). Reduction but no shift in brain activation after arithmetic learning in children: A simultaneous fNIRS-EEG study. *Scientific reports, 8*(1), 1707.

Thomas, G., & Dowker, A. (2000). *Mathematics anxiety and related factors in young children.* Paper presented at the British Psychological Society Developmental Section Conference, Bristol, UK.
